# Secondary structure and ^1^H, ^13^C and ^15^N resonance assignments of Skint-1: a selecting ligand for a murine γδ T cell subset implicated in tumour suppression

**DOI:** 10.1007/s12104-016-9700-0

**Published:** 2016-08-04

**Authors:** M. Salim, C. R. Willcox, F. Mohammed, A. C. Hayday, M. Overduin, B. E. Willcox, T. J. Knowles

**Affiliations:** 1 Cancer Immunology and Immunotherapy Centre, Institute of Immunology and Immunotherapy, University of Birmingham, Edgbaston, Birmingham, B15 2TT UK; 2 Francis Crick Institute, Lincoln’s Inn Fields Research Laboratories, London WC2A 3LY, UK and Peter Gorer Department of Immunobiology, King’s College London, London, UK; 3 Department of Biochemistry, Faculty of Medicine & Dentistry, Edmonton, T6G 2H7 Alberta Canada; 4 Henry Wellcome Building for Biomolecular NMR, School of Cancer Sciences, University of Edgbaston, Birmingham, B15 2TT UK; 5 School of Biosciences, University of Birmingham, Edgbaston, Birmingham, B15 2TT UK

**Keywords:** Skint-1, Dendritic epidermal T cells, γδ T cells, Intraepithelial lymphocytes, NMR, Backbone resonance assignment, Secondary structure

## Abstract

A study describing the ^1^H, ^13^C and ^15^N backbone and side chain chemical shift assignments and secondary structure of Skint-1 a prototypic member of a family of mouse genes, of which Skint-1 is involved in the development of the dendritic epidermal T cell (DETC) subset of γδ T cells.

## Introduction

γδ T cells are a subset of unconventional lymphocytes that are often localised to body surfaces, where they comprise a fraction of intraepithelial lymphocytes (IELs) and are thought to contribute to lymphoid stress surveillance, responding to signs of tissue damage, microbial infection and carcinogenesis (Hayday 2009). Dendritic epidermal T cells (DETC) cells are a well defined skin-resident subset of murine IELs which express an invariant Vγ5Vδ1 T cell receptor (TCR) and have been shown to contribute to immune protection from cutaneous malignancy (Girardi et al. 2001), consistent with a role in tumour immunosurveillance. However, the molecular mechanisms underlying the selection of such IEL populations and how they recognise target cells are ill defined.

Recent studies have identified *Skint*-*1*, a novel gene expressed in the thymus and skin that is the prototypic member of a family of related receptors, as playing a critical role in DETC cell selection (Lewis et al. 2006; Boyden et al. 2008). Expression of truncated Skint-1 led to a selective loss of the DETC γδ T cell subset, thus indicating a vital role for Skint-1 in the development of DETC γδ T cells, and establishing Skint-1 as a potential ligand for the Vγ5Vδ1 TCR (Boyden et al. 2008). Understanding the structural basis of Skint-1 function is likely to shed light on the immunobiology of DETC γδ T cells, including their role in tumour recognition.

In this article we report the ^1^H ^13^C and ^15^N backbone and sidechain assignments, and secondary structure predictions of the membrane distal immunoglobulin (Ig) domain of Skint-1, which has previously been shown to be critical for Skint-1 function (Barbee et al. 2011). The full Ig domain consists of 119 residues. In combination with parallel mutational studies (Salim et al. JBC in press), the results should help define the key molecular features of Skint-1 in DETC selection, and will inform structural studies of other Skint family members.

## Methods

The recombinant Skint-1 DV residues S24 to T141 (without the Immunoglobulin constant domain and transmembrane spanning region) was overexpressed in *Escherichia coli* BL21 (DE3) strain using an ampicillin resistant pET23a vector. The cells were grown to an OD_600_ = 0.6 in M9 minimal medium at 37 °C containing ampicillin, ^15^N-ammonium sulphate and ^13^C-glucose as exclusive nitrogen and carbon source, respectively. Induction was performed at 18 °C using 1 mM IPTG for 16 h. The bacterial cell pellet was then harvested by centrifugation for 20 min at 6000×g. The bacterial pellet was resuspended in phosphate buffered saline and lysed by sonication using a Misonix sonicator 3000 (45 repetitions, 1 on 1 s off). Skint-1 DV inclusion bodies were recovered by centrifugation for 15 min at 75,000×g. The pellet was washed three times in a Triton wash buffer [0.5 % Triton X-100 (v/v), 200 mM NaCl, 10 mM EDTA, 0.01 % Na Azide and 50 mM Tris–HCl pH 8.0]. A final wash without Triton was followed by solubilisation of the protein pellet into 8 M urea containing buffer. Skint-1 DV (30 mg) was renatured by using a dilution refolding method consisting of addition of Skint1 IgV protein drop wise to 1 L refolding buffer containing 5 M urea, 100 mM Tris, 0.4 M l-arginine-HCl, 2 mM EDTA, 0.5 mM oxidised glutathione, 5 mM reduced glutathione and 0.1 mM PMSF, pH 8.3 at 4 °C overnight. The refolding mixture was then dialysed against 100 mM urea overnight and then dialysed for a final time in 100 mM urea and 10 mM Tris pH 8. The refolding mixture was concentrated and purified by size exclusion chromatography using a Superdex-200 (GE Healthcare) column pre-equilibrated with 50 mM NaCl, 20 mM MES pH 6.5. The Skint-1 DV elution profile corresponded to a monomeric state in solution.

NMR experiments were performed at 303 K on Varian Inova 600 and 800 MHz NMR spectrometers equipped with triple resonance cryogenic probes and z-axis pulse field gradients. Skint-1 DV was at a concentration of 1.4 mM in MES pH 6.5 and 50 mM NaCl. Spin system and sequential assignments were made from BEST ^1^H, ^15^N-HSQC, CBCA(CO)NH, HNCACB, HNCA, HN(CO)CA, HNCO, HN(CA)CO and standard H(C)CH TOCSY, (H)CCH TOCSY, ^15^N-edited NOESY-HSQC (τ_mix_ = 100 ms) and ^13^C-edited NOESY-HSQC experiments (τ_mix_ = 100 ms) (Muhandiram and Kay 1994; Schanda et al. 2006; Lescop et al. 2007). Asn and Gln side chain ^1^H and ^15^N resonances were assigned using 3D ^15^N edited NOESY-HSQC and 3D CBCA(CO)NH spectra. All spectra were processed using NMRPipe (Delaglio et al. 1995) and analysed using SPARKY (Goddard TD 2004).

## Assignments and discussion

**Fig. 1:**
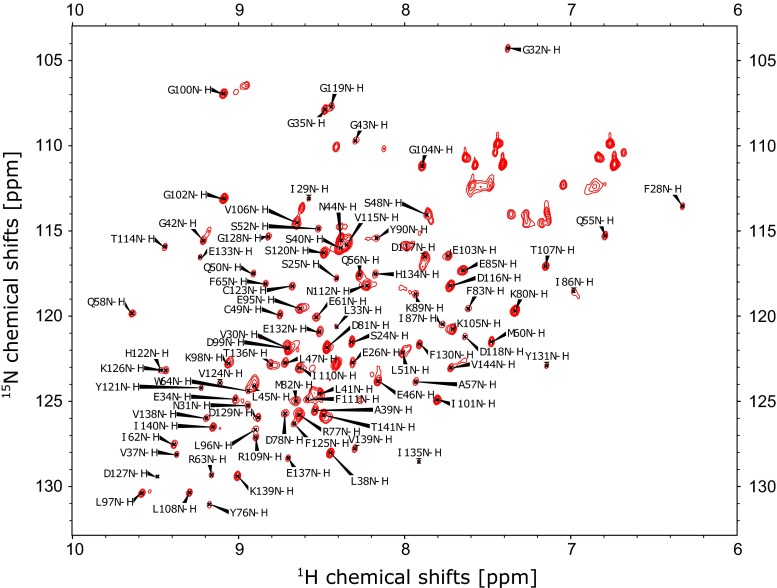
^1^H^15^N-HSQC spectrum of Skint-1 DV domain in 20 mM MES pH 6.5, 50 mM NaCl collected at 298 K on a Varian 800 MHz spectrometer. Backbone ^1^H ^15^N peaks are labelled with their residue assignments

**Fig. 2:**
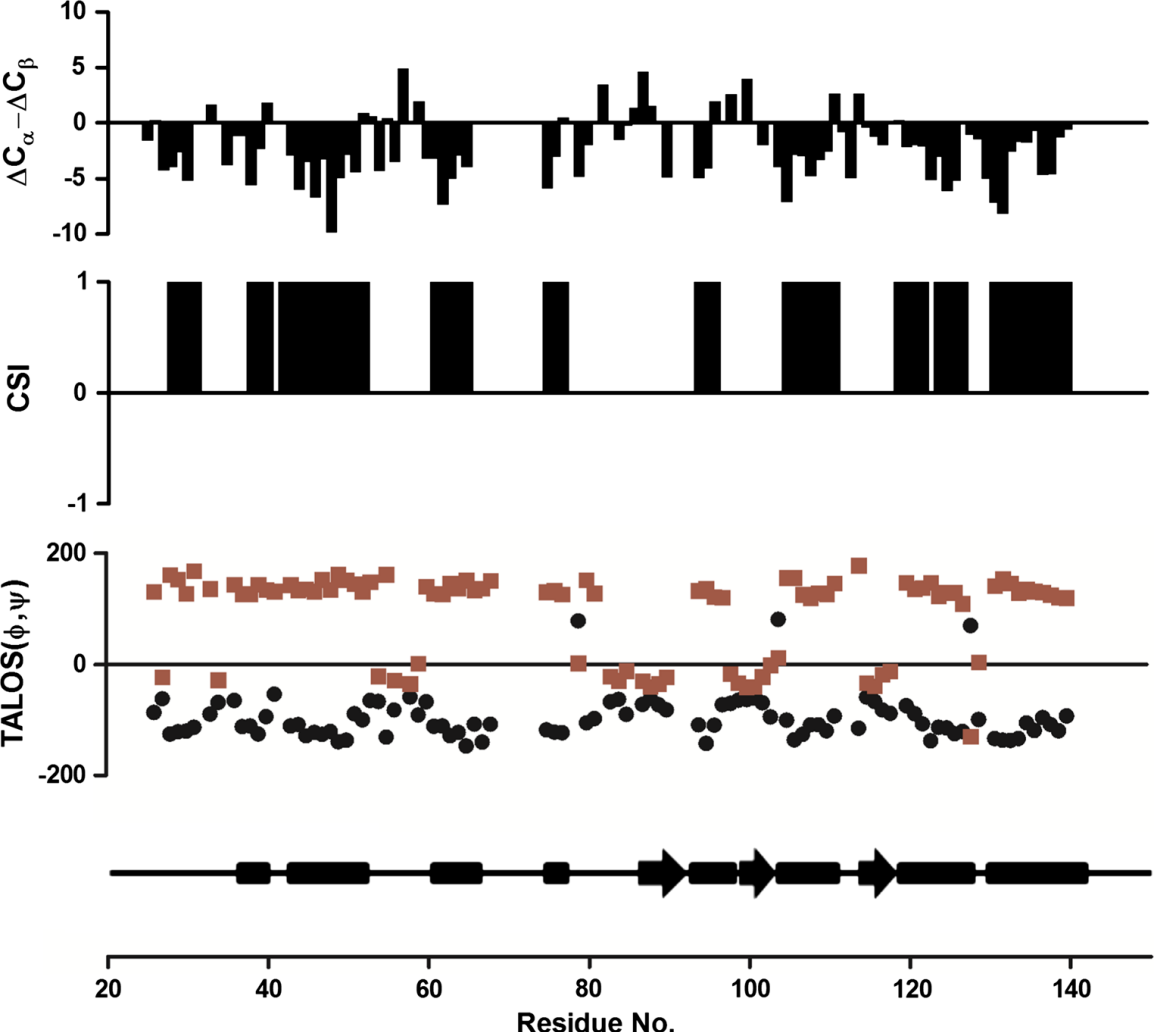
Summary of secondary structure predictions of Skint-1 DV domain with (ΔC^α^ − ΔC^β^), CSI and TALOS results plotted. In the consensus CSI (Wishart and Sykes 1994), the values “1” represent Beta strands while “−1” represent α helical tendency. Backbone dihedral angles (phi, psi) were calculated using TALOS+ (Shen et al. 2009). Phi and Psi are shown in *black* and *red*, respectively. Predicted secondary structure elements are display using *arrows* for α helices and *rectangles* for β strands

The ^1^H, ^13^C, ^15^N HSQC of Skint-1 DV protein is shown (Fig. 1). Considerable linewidth variability was observed. Attempts were made to improve this by varying buffer conditions and temperature but no further improvements could be made. Under the optimal conditions identified, backbone assignments were completed for all non-proline ^1^H, ^13^C and ^15^N backbone resonances except for the amides of M23, H59, G79, S88, and those in the regions 65–74 and 91–93 presumably due to the dynamic nature of these residues resulting in broadening of their chemical shifts, as in many cases no sign of the chemical shifts were observed. All C^β^ resonances have been assigned for all backbone assigned residues. The majority of the side chain resonances of Skint-1 were identified by the analysis of the H(C)CH-TOCSY, (H)CCH-TOCSY and ^13^C-edited NOESY spectra. In total 88.2 % of the backbone, 79.3 % of the sidechains and 60.7 % of the aromatic resonances were assigned. The ^1^H and ^15^N resonances for the NH2 side chains for 3 out of 4 Asn and 4 Gln were assigned completely. No assignments were made for the labile guanidine moiety of Arg, the sidechain NH_3_^+^ of Lys, and the side chain carboxyl groups of Asp and Glu. In total 13 chemical shifts were identified outside the normal range: HD11/13 of L51 (−0.594 ppm); HB3 of L74 (−0.194); HB2, HB3, HG & HD of L108 (0.286, −0.801, 0.0270, −0.207 ppm, respectively); HB3 of H122 (1.180 ppm); HB3 C123 (0.622 ppm); CB of E132 (36.503 ppm); CB of E133 (36.291); HA of E133 (6.023 ppm) and HG13 of I135 (−0.751 ppm). Of these chemical shifts, all except those of L74 are within β-sheet residues (Fig. 2 and discussed below) and presumably within the hydrophobic core of the protein, whilst L74 is just prior to the start of the fourth β-strand and likely in a similar environment.

The secondary structure of Skint-1 DV was predicted using the chemical shift difference method between measured values and random-coil values of C^α^, C^β^ and (ΔC^α^ − ΔC^β^) based on the TALOS (Cornilescu and Bax 1999) and CSI (Wishart and Sykes 1994) protocols. The comparison between folded and random coil chemical shifts show that C^α^ resonances tend to shift upfield in β-sheets and extended stands, but tend to shift downfield in α-helices, as for the C^β^ resonances the reverse is true. As the C^α^ and C^β^ secondary shifts are of similar magnitude and opposite sign for both helices and shifts, subtraction of the C^α^ and C^β^ secondary shifts (ΔC^α^ − ΔC^β^) enhances the correlation between the secondary structural elements and the secondary shifts. Based on the (ΔC^α^ − ΔC^β^), TALOS and CSI plots there are 3 α-helices and 8 β-strands of at least 3 amino acids in length (Fig. 2); the α-helices were estimated to encompass residues I87 to V91, K98 to E103 and V115 to D118 whilst the β-strands span residues V37 to A39, G43 to L51, E61 to F65 and L75 to Y76, R93 to L97, G104 to I110, G119 to D127 and F130 to T141.

The chemical shift values for the ^1^H, ^13^C and ^15^N resonances of the Skint-1 DV have been deposited in the BioMagResBank (http://www.bmrb.wisc.edu) under accession number 17833.
